# Behandlungsregime tiefer sternaler Wundinfektionen nach kardiochirurgischen Eingriffen im interdisziplinären Ansatz

**DOI:** 10.1007/s00113-023-01394-x

**Published:** 2023-12-12

**Authors:** D. Bieler, A. Franke, M. Völlmecke, S. Hentsch, A. Markewitz, E. Kollig

**Affiliations:** 1 Klinik für Unfallchirurgie und Orthopädie, Wiederherstellungs- und Handchirurgie, Verbrennungsmedizin, Bundeswehrzentralkrankenhaus Koblenz, Rübenacherstraße 170, 56072 Koblenz, Deutschland; 2 Klinik für Orthopädie und Unfallchirurgie, Universitätsklinikum Düsseldorf, Düsseldorf, Deutschland; 3 Bendorf, Deutschland

**Keywords:** Sternotomie, Interdisziplinäre Behandlung, Osteosynthese, Outcome, Komorbiditäten, Sternotomy, Interdisciplinary approach, Osteosynthesis, Outcome, Comorbidities

## Abstract

Ziel dieser Arbeit ist es, anhand der diagnostischen und therapeutischen Herangehensweise bei der interdisziplinären Behandlung von 54 Patienten mit einer tiefen sternalen Wundinfektion (TSWI) nach kardiochirurgischem Eingriff sowie der erzielten Ergebnisse die Wertigkeit eines strukturierten und situationsadaptierten Vorgehens vorzustellen. Es handelte sich um 41 Männer und 13 Frauen mit einem Durchschnittsalter von 65,1 Jahren, die im Zeitraum 2003–2016 nach kardiochirurgischem Eingriff eine TSWI entwickelten. Die Behandlungsstrategie setzte sich zusammen aus dem konsequenten Débridement, einschließlich der Entfernung des einliegenden Fremdmaterials, der Rekonstruktion nach Infektbeherrschung mit stabiler Reosteosynthese und ggf. situationsbedingter Lappenplastik für eine gut durchblutete Defektdeckung und zwingender Vermeidung von Totraum. Es wurden insgesamt 146 Operationen erforderlich (durchschnittlich 2,7 Operationen/Patient, Bereich: eine bis 7 Operationen). In 24,1 % der Fälle konnte ein einzeitiges Vorgehen durchgeführt werden. Bei 41 Patienten wurde zur Wundkonditionierung die „negative pressure wound therapy“ (NPWT) mit programmierten Schwammwechseln angewendet (im Mittel 5 Wechsel, Standardabweichung [SD] ± 5,6 Wechsel über 22 Tage, SD ± 23,9 Tage, Wechselintervall alle 3 bis 4 Tage in 40,7 % der Fälle). Bei 33 Patienten wurde ein beidseitiger myokutaner Pectoralis-major-Lappen genutzt, bei 4 Patienten ein „Vertical-rectus-abdominis-myocutaneous“(VRAM)-Lappen, bei 7 Patienten beide. Am Sternum wurden 43 Osteosynthesen mit winkelstabilen Titanplattensystemen durchgeführt. Sieben Patienten verstarben unter intensivmedizinischer Behandlung (Gesamtmortalität 13 %; *n* = 5 (9,3 %) ≤ 30 Tage) oder im weiteren Verlauf. Mit saniertem Infekt konnten 47 Patienten entlassen werden (87,1 %). Bei 2 Patienten wurden die Implantate nach 2 Jahren wegen Auslockerung entfernt.

## Abstract (en)

The aim of this article is to present the importance of a structured and situation-adapted approach based on the diagnostic and therapeutic strategy in the interdisciplinary treatment of 54 patients with deep sternal wound infections (DSWI) after cardiac surgical interventions and the results achieved. The patients were 41 men and 13 women with an average age of 65.1 years, who developed a DSWI after a cardiac surgical intervention during the period 2003–2016. The treatment strategy included a thorough debridement including the removal of indwelling foreign material, the reconstruction with a stable re-osteosynthesis after overcoming the infection and if necessary, situation-related surgical flaps for a defect coverage with a good blood supply and mandatory avoidance of dead spaces. A total of 146 operations were necessary (average 2.7 operations/patient, range 1–7 operations). In 24.1 % of the cases a one-stage approach could be carried out. In 41 patients negative pressure wound therapy (NPWT) with programmed sponge changing was used for wound conditioning (mean 5 changes, standard deviation, SD± 5.6 changes over 22 days, SD± 23.9 days, change interval every 3–4 days in 40.7% of the cases). In 33 patients a bilateral myocutaneous pectoralis major flap was used, in 4 patients a vertical rectus abdominis myocutaneous (VRAM) flap and in 7 patients both were carried out. A total of 43 osteosynthesis procedures were carried out on the sternum with fixed-angle titanium plates. Of the patients 7 died during intensive care unit treatment (total mortality 13 %, *n* = 5, 9.3 % ≤ 30 days) or in the later course. Of the patients 47 (87.1 %) could be discharged with a cleansed infection. In 2 patients the implant was removed after 2 years due to loosening.

## Einführung

Die mediane Sternotomie ist der am häufigsten gewählte operative Zugangsweg in der Kardiochirurgie. Die Readaptation der Sternumhälften ist nicht durchweg erfolgreich; es etablieren sich Instabilitäten mit und ohne Infektion. Die publizierten Raten der tiefen, sternalen Wundinfektion (TSWI) bewegen sich zwischen 0,6 und 16 % [1–5]. Wie ernst diese Komplikation zu werten ist, belegen Mortalitätsraten bis zu 25 % [6–8].

Zur Diagnose einer Poststernotomiemediastinitis können die Kriterien der Centers for Disease Control and Prevention (CDC) aus dem Jahr 1999 und/oder die publizierten Kriterien nach El Oakley und Wright herangezogen werden [9, 10]. Es wurden in den vergangenen Jahren verschiedene Algorithmen zur Beherrschung und Sanierung der TSWI und auch für die Rekonstruktion von resultierenden sternalen Defekten publiziert [8, 11]. Ein evidenzbasierter Goldstandard ist jedoch nach wie vor nicht etabliert. Definiert wurden zwar die Kerninhalte eines erfolgversprechenden Vorgehens (Tab. 1), aber nicht die Zuständigkeiten und die Möglichkeiten der einzelnen beteiligten Fachdisziplinen.

**Table 1:** 

Postulierte Kerninhalte eines erfolgversprechenden Vorgehens bei TSWI
Komplette Entfernung des Fremdmaterials
Konsequente Nekrosektomie von Knochen und Weichteilen
Infektsanierung, flankiert durch resistogrammgerechte Antibiotikabehandlung, optional knochengängig
Wundkonditionierung mithilfe temporärer Deckung durch NPWT
Knöcherne Defektauffüllung mit autologem Spongiosatransplantatknochen
Stabile Osteosynthese zur Wiederherstellung der Thorax-Compliance und zur Ausbildung einer stabilen Narbe
Spannungsfreie Deckung mit gut durchblutetem Weichgewebe

*NPWT* „negative pressure wound therapy“

Mortalitätsraten bis zu 25 % belegen die Ernsthaftigkeit der tiefen, sternalen Wundinfektion

Da es sich bei den Betroffenen um Hochrisikopatienten handelt, die systemisch belastet sind, erklären sich zum einen die hohen Mortalitätsraten [1, 2]. Zum anderen ist eine erfolgreiche Behandlung nur erreichbar, wenn alle beteiligten Fachdisziplinen aus Patientenperspektive jeweils ihre speziellen Kompetenzen einbringen.

Mit der im Folgenden vorgestellten, retrospektiven Auswertung sollen das interdisziplinäre Management und die Resultate der Behandlung der TSWI aus einem 11-Jahres-Zeitraum (2005–2016) vorgestellt werden. Insbesondere stehen die erforderlichen Ressourcen, das Erregerspektrum und das definitive Vorgehen mit einer stabilen Plattenosteosynthese unter einer gut durchbluteten Weichgewebsdeckung im Fokus dieser retrospektiven Analyse.

## Material und Methode

### Patientenkollektiv

Im Zeitraum von 2005 bis 2016 wurden 54 Patienten mit einer etablierten TSWI nach kardiochirurgischem Eingriff interdisziplinär behandelt. Das Patientenkollektiv rekrutierte sich aus der eigenen Klinik (*n* = 37, 68,5 %) sowie durch zuverlegte Patienten (*n* = 17, 31,5 % der Fälle).

In Anlehnung an die CDC-Kriterien [10] wurde die Diagnose einer TSWI gestellt, wenn mindestens 2 der folgenden Kriterien erfüllt waren:

- klinische Parameter:
  - eitrige Sekretion,
  - Nekrosen,
  - purulente Wunddehiszenz,
  - Fieber,
  - palpatorische Instabilität des Sternums,
- pathologisch erhöhte laborchemische Parameter (Blutbild [BB], C‑reaktives Protein [CRP], Prokalzitonin [PCT]),
- kultureller *oder* histologischer Keimnachweis im Wundsekret.

Ausgeschlossen wurden Patienten mit oberflächlichen Infekten, die nach einmaliger Weichgewebsrevision oder ohne operative Intervention erfolgreich zur Ausheilung gebracht wurden. Die Behandlungsunterlagen wurden hinsichtlich typischer und bekannter Risikofaktoren evaluiert und anonymisiert erfasst. Die Patientencharakteristika mit den demografischen Angaben sind in Tab. 2 aufgeführt.

**Table 2:** 

Arbeitsgruppe	Patienten mit Sternotomie	TSWI	Verursachende Erreger (Anteil, %)					
		Anzahl, *n* (Anteil, %)	*Staph. epidermidis*	*Staph. aureus*	MRSA	Gramnegative	MRGN/VRE	Mischinfektionen, grampositiv und -negativ
Baillot et al. (2010; [1])	23.999	267 (1,1)	45	–	4,9	unbekannt	–	–
Morisaki et al. (2011; [7])	2720	59 (2,2)	–	–	52,5	13,6	–	–
Eyiletin et al. (2009; [12])	4996	52 (1,04)	29,2	6,2	23,1	30,8	–	6,2
Kobayashi et al. (2011; [25])	741	16 (2,2)	12,5 (MRSE)	6,25	68,75	12,5	–	–
Tocco et al. (2009; [27])	–	21	33,3 (MRSE)	23,8	14,3	47,6	–	52,4
Eigene Studiengruppe	5703	*n* = 37 (0,64 %) *n* = 54 (0,95 %)	42,6 (*n* = 23)	20,4 (*n* = 9)	5,6 (*n* = 3	55,6 (*n* = 30)	1,9 (*n* = 1)	18,5 (*n* = 10)

*MRGN* multiresistente gramnegative Bakterien, *MRSA* Methicillin-resistenter *Staphylococcus aureus, TSWI* tiefe sternale Wundinfektion, *VRE* Vancomycin-resistenter *Enterococcus*, *MRSE* Methicillin-resistenter *Staphylococcus epidermidis*

### Präoperative Maßnahmen

#### Erregerdiagnostik.

Aus der Wunddehiszenz wurden vor der geplanten Revision mikrobiologische Abstriche entnommen. Zusätzliche mikrobiologische Untersuchungen wurden an Gewebeproben, die bei der operativen Revision gewonnen wurden, durchgeführt. Je nach klinischer Situation und Sekretmenge wurden im Verlauf, insbesondere unter der „negative pressure wound therapy“(NPWT), weitere mikrobiologische Proben zum Ausschluss einer Änderung der Keimbesiedelung der Wunde entnommen und untersucht.

#### Systemisches Infektionsmonitoring.

Die Serumparameter C‑reaktives Protein (CRP) und Prokalzitonin (PCT) dienten dem systemischen Infektionsmonitoring, wobei dem klinischen Befund die höhere Gewichtung hinsichtlich der therapeutischen Entscheidungsfindung eingeräumt wurde.

#### Diagnosesicherung und Operationsplanung.

**Figure 1:**
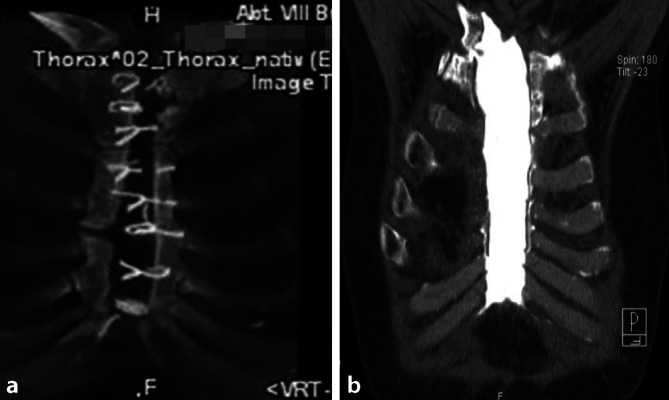

Zur Sicherung der Diagnose und Operationsplanung wurde regelhaft eine Computertomographie des Thorax angefertigt, um die knöcherne Situation am Brustbein individuell zu beurteilen (Abb. 1).

### Operatives Vorgehen

Die Infektsanierung hatte oberste Priorität. Die konsequente operative Entfernung jeglichen Fremdmaterials und des nekrotischen Gewebes, einschließlich des avitalen oder deperiostierten Knochens, stellt eine Conditio sine qua non dar. Das Débridement wurde fortgesetzt, bis an allen Stellen vitales Gewebe und Knochen resultierte. Der chirurgisch gesäuberte Wundgrund wurde anschließend zunächst über eine NPWT konditioniert. Die NPWT-Verband-Anordnung wurde routinemäßig 3 bis 5 Tage nach der Anlage gewechselt; der Unterdruck wurde kontinuierlich bei 75 bis 100 mm Hg reguliert.

Sofern hinreichend Restmasse am Sternum vorhanden war, erfolgte eine Osteosynthese mit zentral entkoppelbaren, am Korpus des Sternums und auf den Rippenansätzen verankerten winkelstabilen Titanplatten (Sternum-Fixationssystem; Fa. DePuy-Synthes GmbH, Oberdorf, Switzerland). In allen Fällen eines rekonstruierbaren Sternums erfolgte die Augmentation mithilfe einer additiven autologen Spongiosaplastik vom Becken und autologem, plättchenreichen Plasma (PRP). Bei ausgedehntem Knochendefekt des Brustbeins wurden nach der Transplantation von Spongiosablöcken winkelstabile Implantate desselben Systems mit seitlicher langstreckiger Verlängerung auf die Rippen eingesetzt, um eine adäquate, knöcherne Verankerung und Krafteinleitung zu gewährleisten.

Unabdingbar ist die operative Entfernung jeglichen Fremdmaterials und des nekrotischen Gewebes

**Figure 2:**
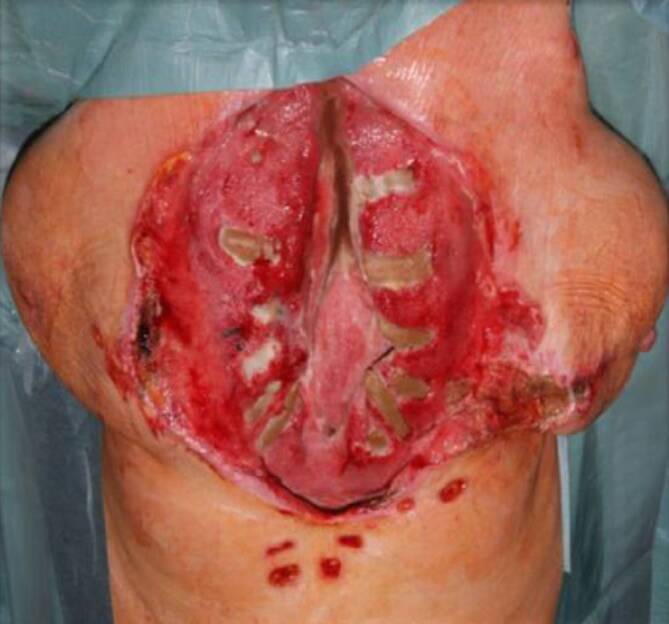

Bei großen Weichteildefekten kam entweder der beidseitige, myokutane Pectoralis-major- oder ein „Vertical-rectus-abdominis-myocutaneous“(VRAM)-Lappen zur Anwendung. Ein wesentliches Ziel der VRAM-Lappenplastik war die Beseitigung jeglichen Totraums bei spannungsfreiem Weichgewebsverschluss. Im Fall eines vollständig verlorenen Sternums wurde als Ersatz und vitaler Platzhalter ein Rectus-abdominis-Lappen eingesetzt und hierüber eine horizontale Osteosynthese auf die knöchernen Rippenanteile beidseits (Abb. 2, 3 und 4) aufgebracht.

**Figure 3:**
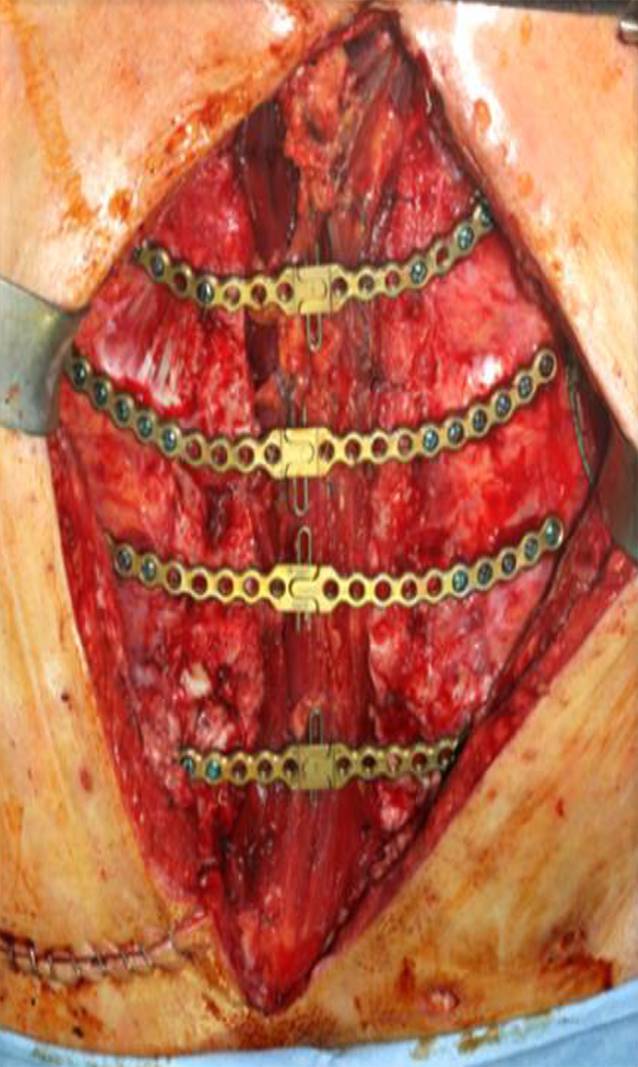

**Figure 4:**
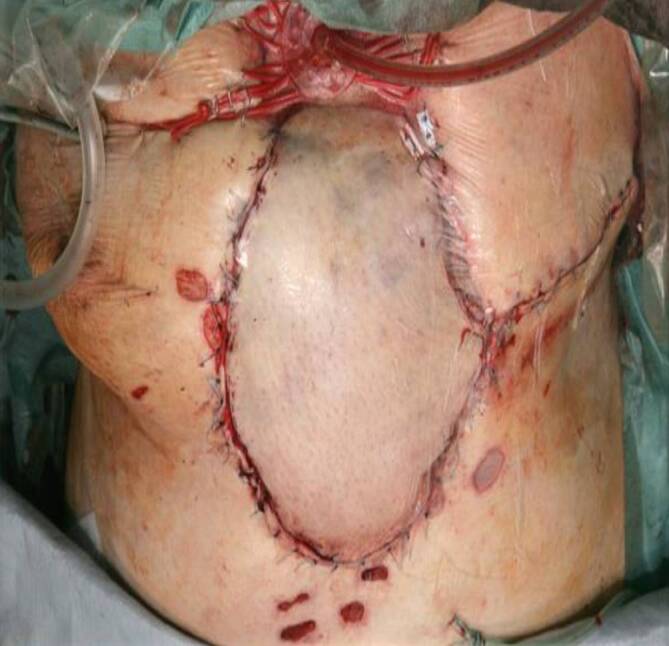

Eine freie Lappenplastik oder eine Omentum-majus–Plastik wurde nicht verwendet, obwohl Letztere grundsätzlich mit in Betracht gezogen wurde. In der interdisziplinären Entscheidungsfindung wurde wegen der additiven Systembelastung durch einen Eingriff in die Bauchhöhle bei den individuellen Risikoprofilen der Patienten davon Abstand genommen.

## Ergebnisse

Bei den 54 Patienten mit einem mittleren Alter von 65,1 Jahren Standardabweichung [SD]± 10,2 Jahre) handelte es sich um 41 Männer und 13 Frauen mit einem durchschnittlichen Body-Mass-Index (BMI) von 31,2 kg/m^2^ (SD ± 4,9 kg/m^2^). Einen Überblick über die epidemiologischen Daten, bekannte Nebenerkrankungen und Confounder gibt Tab. 3.

**Table 3:** 

Parameter		Wert
Patienten	Eingeschlossen	54
	♂ (Anzahl, *n* [Anteil, %])	41 (76)
	♀ (Anzahl, *n* [Anteil, %])	13 (24)
	Alter (Jahre)	
	– M ± SD	65,1 ± 10,2
	– Min–Max	44–88
Risikofaktoren	Body-Mass-Index (kg/m^2^)	
	– M ± SD	31,2 ± 4,9
	– Min–Max	23–44
	DM (Anzahl, *n* [Anteil, %])	30 (56)
	IDDM (Anzahl, *n* [Anteil, %])	11 (20)
	Rauchen (Anzahl, *n* [Anteil, %])	27 (50)
	COPD (Anzahl, *n* [Anteil, %])	15 (28)
	pAVK (Anzahl, *n* [Anteil, %])	10 (19)
	Bluthochdruck (Anzahl, *n* [Anteil, %])	49 (91)
	Nierenversagen (Anzahl, *n* [Anteil, %])	10 (19)
Medizinische Vorgeschichte	Akute Angina pectoris (Anzahl, *n* [Anteil, %])	49 (91)
	NSTEMI, bei Aufnahme (Anzahl, *n* [Anteil, %])	6 (11)
	STEMI, bei Aufnahme (Anzahl, *n* [Anteil, %])	4 (7)
	Z. n. MI (Anzahl, *n* [Anteil, %])	23 (43)
	Z. n. PTCA (Anzahl, *n* [Anteil, %])	12 (22)
	Z. n. TIA (Anzahl, *n* [Anteil, %])	8 (15)

*DM* Diabetes mellitus, *IDDM* insulinabhängiger Diabetes mellitus, *COPD* „chronic obstructive pulmonary disease“, *M* Mittelwert, *NSTEMI* Nicht-ST-Strecken-Elevationsmyokardinfarkt, *pAVK* periphere arterielle Verschlusskrankheit,* STEMI* ST-Strecken-Elevationsmyokardinfarkt, *MI* Myokardinfarkt, *PTCA* perkutane transluminale Koronarangioplastie, *SD* Standardabweichung, *TIA* transitorische ischämische Attacke

Bezogen auf die initiale kardiochirurgische Versorgung wurden 49 Patienten (90,7 %) bei instabiler Angina pectoris dringlich operiert. Die Operationsdauer für die Erstversorgung betrug 210 min (SD ± 49 min). Bei 5 Patienten (9,1 %) wurde beidseits die A. mammaria als Graft verwendet.

Es wurden insgesamt 146 operative Revisionen bei den 54 Patienten mit TSWI durchgeführt (durchschnittlich 2,7, SD ± 1,4, Min–Max 1–7, eine Revision in 24,1 % der Fälle; 2 Revisionen in 25,9 % der Fälle; 3 Revisionen in 22,2 % der Fälle; 4 Revisionen in 14,8 % der Fälle; 5 Revisionen in 11,1 % der Fälle und 7 Revisionen in 1,9 % der Fälle). Es konnten bei 39 Patienten (72,7 % der Fälle) ein Keimwachstum gesichert und die in Tab. 4 dargestellten Erreger identifiziert werden. Führender Keim war mit 42,6 % der Fälle Staph. epidermidis, gefolgt von *Enterococcus faecalis* in 24,1 % der Fälle und *Staph. aureus* (einschließlich Methicillin-resistentem *Staphylococcus aureus* [MRSA] 13 % der Fälle; Tab. 4).

**Table 4:** 

Nachgewiesene Erreger	Anzahl (*n*)	Anteil (%)
*Staph. epidermidis*	23	42,6
*Enterococcus faecalis*	13	24,1
*Staph. aureus*	9	16,6
*Pseudomonas aeruginosa*	7	13,0
Koagulase-neg. *Staphylococcus*	5	9,3
*Klebsiella*-Spezies	3	5,6
*E. coli*	3	5,6
MRSA	3	5,6
*Enterococcus cloacae*	2	3,7
*Enterobacter aerogenes*	1	1,9
*Clostridium difficile*	1	1,9
*Proteus mirabilis*	1	1,9

*MRSA* Methicillin-resistenter Staphylococcus aureus, multipler Erregernachweis möglich

In 7,4 % der Fälle lagen multiresistente Erreger vor (3-mal MRSA, einmal Vancomycin-resistenter Enterococcus [VRE]). In 15 Fällen konnte mikrobiologisch kein Keim angezüchtet werden; die Antibiotikabehandlung konnte in diesen Fällen nur kalkuliert empirisch mit einer Breitspektrumsubstanz erfolgen oder wurde entsprechend an der nebenbefundlich dokumentierten Besiedelung/Kontamination von Urin, Blutkultur, Katheter- und/oder Fremdmaterial ausgerichtet.

Bei 13 Patienten (24,1 % der Fälle) konnte eine einzeitige Sanierung erfolgen. Voraussetzungen waren ein radikales Débridement avitaler Gewebe- und Knochenanteile, kein verbliebener Totraum nach Osteosynthese und ein spannungsfreier Wundverschluss. Die Weichteildeckung erfolgte in diesen Fällen mithilfe der beidseitigen, myokutanen Pektoralislappenplastik. In diesen 13 Fällen blieb die mikrobiologische Diagnostik ohne Keimnachweis.

Waren die Voraussetzungen für eine einzeitige Sanierung nicht gegeben, erfolgte zunächst eine konditionierende lokale NPWT. Dies wurde bei 41 Patienten (75,9 % der Fälle) erforderlich. Die programmierten Wechsel (4,9/Patient, SD ± 5,6 Wechsel [Maximum 21 Wechsel]) fanden in 40,7 % der Fälle zwischen dem 3. und 4. Tag statt. Die Gesamtdauer der NPWT betrug im Mittel 22 Tage, SD ± 23,9 Tage mit einem kontinuierlichen Unterdruck von 75–100 mm Hg. Eine definitive Versorgung wurde bei Vorliegen von 3 negativen Wundabstrichen und granulierendem, vitalen Wundgrund geplant.

Bei 10 Patienten (18,5 % der Fälle) war eine Wund- und Weichteilrevision mit plastischer Deckung ausreichend, und die primäre Osteosynthese mit der Verdrahtung des Sternums konnte belassen werden. Eine Reverdrahtung erfolgte in einem Fall. Es erhielten 44 Patienten (81,5 %) eine winkelstabile Plattenosteosynthese. Bei 11 Patienten (20,4 % der Fälle) boten die Sternumreste kein ausreichendes Plattenlager; in diesen Fällen wurde eine quere, kostokostale Thoraxstabilisierung mit Rippenplatten durchgeführt.

Additiv wurde bei 37 Patienten (68,5 %) autologes PRP lokal eingebracht (ANGEL-System; Fa. Arthrex, München, Deutschland, oder GPS-System; Fa. Biomet Deutschland GmbH, Berlin, Deutschland). Eine autologe Spongiosaplastik vom Beckenkamm wurde bei 33 Patienten zur knöchernen Defektfüllung eingebracht.

Zur plastischen, spannungsfreien und gut durchbluteten Deckung sowie zur Auffüllung von verbleibendem Totraum mit vitalem Gewebe kam die beidseitige, myokutane Pectoralis-major-Lappenplastik in 33 Fällen zur Anwendung; in 7 Fällen wurde ein VRAM-Lappen von rechts zusätzlich eingeschwenkt, da bei diesen Patienten die rechtsseitige A. mammaria interna nicht für die primäre Operation verwendet worden war. In 4 Fällen wurde ein isolierter VRAM-Lappen mit defektdeckender Hautspindel von rechts ohne Pektoralisverschiebelappenplastik verwendet.

Bei komplett verlorenem Sternum wurde in 6 Fällen als Ersatz und vitaler Platzhalter ein Rectus-abdominis-Lappen eingesetzt, hierüber eine horizontale Osteosynthese auf die verbliebenen knöchernen Rippenanteile beidseits (Abb. 3).

In einem Fall wurde der VRAM-Lappen als myokutaner Lappen bei einem ausgedehntem Weichgewebsdefekt, der durch die beidseitige Pectoralis-major-Lappenplastik nicht mehr zu decken war, transponiert (Abb. 4).

Bei 4 Patienten (7,4 % der Gesamtgruppe und 9,3 % der plattenosteosynthetisch versorgten TSWI) musste eine operative Revision lokal durchgeführt werden. Hier erfolgten in allen Fällen die einzeitige Revision und direkte Reosteosynthese mit winkelstabilen Platten.

Alle 47 überlebenden Patienten mit einer TSWI konnten mit abgeheilter, infektionsfreier Wundsituation aus der stationären Behandlung entlassen werden.

Alle 47 überlebenden Patienten wurden mit abgeheilter, infektionsfreier Wundsituation entlassen

In 2 Fällen wurde wegen einer nativradiologisch nachgewiesenen Auslockerung der Osteosynthesen die Implantatentfernung durchschnittlich 24 Monate nach der Restabilisierung erforderlich. Eine Infektionsrekurrenz war nicht zu verzeichnen.

Im Mittel verbrachten die Patienten 44 Tage (SD ± 23 Tage) im Krankenhaus und 6 Tage (SD ± 2 Tage) auf der Intensivstation. Transfusionspflichtig waren 24 Patienten, verabreicht wurden im Mittel 3,2 Erythrozytenkonzentrate und 3,7 Fresh-Frozen-Plasma-Konzentrate bei 3 Patienten. Die 30-Tage-Mortalität betrug 13 % (7 von 54).

## Diskussion

Die TSWI nach kardiochirurgischem Eingriff stellt eine ernste Komplikation dar. Die Mortalitätsrate wird auch unter modernen Behandlungsregimes mit bis zu 25 % angegeben [7, 8]. In der hier vorgestellten Studiengruppe betrug die Mortalitätsrate 13 %.

### Risikofaktoren für die Entstehung postoperativer Infektionen nach einer Sternotomie

Die inzwischen nachgewiesenen und häufig in Kombination auftretenden Risikofaktoren für die Entstehung postoperativer Infektionen nach einer Sternotomie bei kardiochirurgischen Eingriffen sind in der nachfolgender Synopsis zusammengefasst [1, 2, 12, 13]:

- Lebensalter,
- BMI > 30 kg/m^2^,
- Diabetes mellitus,
- „chronic obstructive pulmonary disease“ (COPD),
- chronisches Nierenversagen,
- prolongierte Intubation und Beatmung,
- Reoperation wegen Blutung,
- beidseitige Verwendung der A. mammaria interna,
- langfristige Kortisonmedikation,
- iatrogene Immunsuppression,
- begleitende Infektion,
- geringe Herzauswurfleistung.

Gemäß Tab. 3 konnte im untersuchten Patientenkollektiv ein vergleichbares Risikoprofil dokumentiert werden.

### Interdisziplinäre Therapie der tiefen sternalen Wundinfektion

Die Therapie einer TSWI, die in den letzten Jahren einen Paradigmenwechsel erfahren hat, stützt sich auf die folgenden Behandlungspfeiler:

- Infektsanierung durch
  - komplette Entfernung von Fremdmaterial,
  - Débridement im Sinne der konsequenten Nekrosektomie von Knochen und Weichteilen,
  - nachfolgende Wundkonditionierung (z. B. Vakuum[VAC]-Therapie) ergänzt durch
  - resistogrammgerechte Antibiotikabehandlung,
  - vitale, spannungsfreie Weichgewebsdeckung resp. Totraumauffüllung mit gut durchblutetem Gewebe (regelhaft Lappenplastiken erforderlich),
  - Restabilisierung des Thorax durch sichere Reosteosyntheseverfahren am Sternum bei infektionsfreiem Situs.

**Figure 5:**
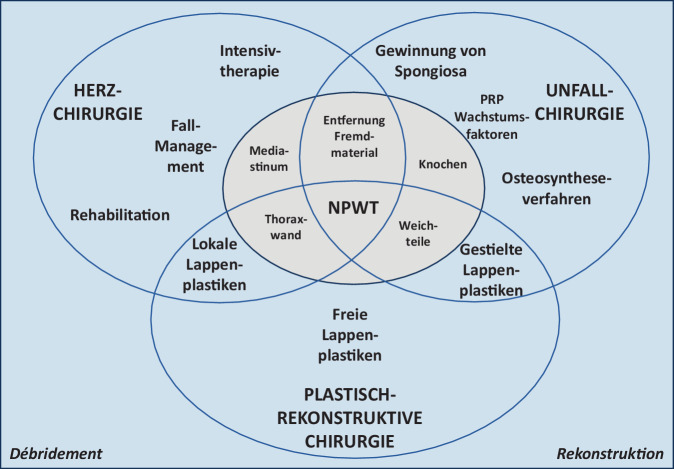

Hierbei ist einem interdisziplinären Vorgehen der Vorzug zu geben, da unterschiedlichste Fachrichtungen (ohne Anspruch auf Vollständigkeit), wie Abb. 5 zeigt, über entsprechende Expertisen verfügen.

Die konsequente Umsetzung der Behandlungspfeiler und -prinzipien im Team ist Conditio sine qua non für den Behandlungserfolg. Die geringe Anzahl der Revisionseingriffe bei TSWI nach Osteosynthese (*n* = 4) im eigenen Vorgehen unterstreicht diese These und spricht für den „team approach“.

#### Infektsanierung

Bei der lokalen Infektsanierung hat neben dem konsequenten Débridement die Unterdruckwundbehandlung die frühere Spül-Saug-Drainagen-Behandlung de facto abgelöst und findet breite Anwendung bei vorliegender TSWI. Baillot et al. [1] konnten in einer Kohortenstudie an 23.499 Sternotomien mit 267 TSWI nachweisen, dass die Mortalitätsrate der TSWI signifikant niedriger ausfiel, nachdem die VAC-Behandlung in das Therapieregime integriert wurde (4,8 % vs. 14,1 %, *p* = 0,01).

Bezüglich der Infektsanierung sind in Tab. 2 die in der Literatur genannten Keime aufgeführt, die bei TSWI nachgewiesen wurden. Es wird deutlich, dass die Multiresistenzen wie die gramnegativen Keime quantitativ und hinsichtlich des Behandlungsaufwands qualitativ zugenommen haben. Auffällig ist weiter der hohe Anteil der Patienten ohne Keimnachweis, der im vorgestellten Patientenkollektiv ebenfalls in 27,8 % der Fälle beobachtet wurde. Hier scheint nicht die primäre Infektion, sondern das anerge bis avitale Gewebe den Defekt zu hinterlassen, mit obligater, sekundärer Besiedelung, die es zu vermeiden gilt.

#### Weichgewebsdeckung resp. Totraumauffüllung

Nach der Infektsanierung und bei noch vorhandenem vitalen Korpus des Sternums erfolgt bei den regelmäßig nach dem konsequenten Débridement einer TSWI resultierenden ausgedehnten Weichgewebsdefekten die Rekonstruktion des Thorax mit einer stabilen Reosteosynthese und lokalen Lappenplastiken zum spannungsfreien Verschluss [14–17]. Aus der Chirurgie an Stütz- und Bewegungsapparat ist hinreichend bekannt, dass insbesondere im Revisionsfall die suffiziente und gut durchblutete Weichgewebsdeckung über einer (Re‑)Osteosynthese die Heilung erst ermöglicht [18–20]. Nach der richtungweisenden Arbeit von Jurkiewicz et al. [21] aus dem Jahr 1980 wurden Lappenplastiken beim Management der TSWI ein unverzichtbarer Teil des therapeutischen Algorithmus. Muskuläre oder myokutane Lappenplastiken gewährleisten die Defektdeckung, die Auffüllung von infektionsbegünstigenden Toträumen und kontrollieren durch die gute Durchblutung den Infekt [22].

Lappenplastiken sind beim Management der TSWI ein unverzichtbarer Teil des Therapiealgorithmus

Der Pectoralis-major-Lappen stellt heute das „Arbeitspferd“ für dieses Problem dar [1, 12, 23–27]. Auch im eigenen Patientengut dominierte die beidseitige Pectoralis-major-Lappenplastik. Alternativ oder ergänzend im plastisch-rekonstruktiven Spektrum wird die M.-rectus-abdominis-Plastik angeführt [28] und als VRAM- oder „Transverse-rectus-abdominis-myocutaneous“(TRAM)-Lappen eingesetzt. Andere Arbeitsgruppen führen die Defektdeckung mit einem Omentum-majus- oder Latissimus-dorsi-Lappen durch [29–32]. Diese kamen beim eigenen Patientengut nicht zur Anwendung, um erstens die Systembelastung durch Eröffnen einer zweiten Körperhöhle gering zu halten und zweitens noch eine Rückzugsmöglichkeit beim Scheitern des Eingriffs zu haben.

Die Vorteile eines algorithmusgesteuerten Vorgehens wurde von der Arbeitsgruppe um Weinand vorgestellt [11, 32]. Hier wurden die Lappenplastiken jeweils der resultierenden Defektgröße angepasst. Mithilfe des Algorithmus gelang eine Reduktion der Wunddehiszenzen um 7 % im Vergleich zu der früheren, nichtalgorithmusgebundenen Vorgehensweise. Signifikant niedriger fiel auch die Dauer der Intensivtherapie aus [11, 32].

#### Restabilisierung des Thorax

Als dritte Säule des erfolgreichen Managements der TSWI wird die Wiederherstellung der Stabilität des Thorax erachtet, da eine persistierende Infektion und Instabilität sich gegenseitig begünstigen. Standardverfahren für die Osteosynthese nach medianer Sternotomie ist die Verdrahtung. Dieses Verfahren ist einfach, schnell zu bewerkstelligen, kostengünstig und hat den Vorteil der raschen Entfernbarkeit im Fall eines Revisionseingriffes. Der wesentliche Nachteil dieser Technik besteht in der geringeren mechanischen Stabilität gegenüber konventionellen Osteosynthesen, wie z. B. einer längerstreckigen queren Plattenanordnung. Dies trifft insbesondere zu, wenn relevante Sternumdefekte vorliegen. Postoperative Instabilitäten bis hin zur Pseudarthrose stellen eine regelmäßig auftretende Komplikation in der elektiven Herzchirurgie dar [2].

Persistierende Infektion und Thoraxinstabilität begünstigen sich gegenseitig

Verschiedene Gruppen haben deshalb die Anwendung von alternativen, zuverlässigen Stabilisierungsverfahren bei Patienten mit erhöhtem Risiko für eine postoperative Instabilität untersucht. Mit Erfolg wurden im Vergleich zur konventionellen Verdrahtung thermoreaktive Nitinolklammern [27, 33], ein spezielles Plattensystem für das Sternum mit 2 ventral zu koppelnden Hakenplatten in querer Montage [34], eine rigide Titanplattenosteosynthese des Sternums [2] und Chrom-Nickel-Bänder [35] verwendet.

Huh et al. [36] berichteten 2008 über die erfolgreiche Anwendung eines speziellen Plattensystems für sekundäre Rekonstruktionen nichtinfizierter, instabiler Sterna bei 13 von 14 Patienten mit vorausgegangenem, kardiochirurgischem Eingriff. Diese Arbeitsgruppe sah Vorteile der horizontalen Osteosynthese von Rippe zu Rippe bei osteopenischem oder fragmentiertem Sternum. Die erstmalige Beschreibung der gelungenen Verwendung transversaler Titanplatten bei einer TSWI erfolgte 2007 durch Plass et al. [37] an 3 Patienten. Die erfolgreiche Anwendung moderner Titanplattensysteme mit zentraler Entriegelungsmöglichkeit wurde inzwischen von mehreren Arbeitsgruppen bestätigt [24, 38]. Sie sind bei schlechter Knochenqualität der Verdrahtung hinsichtlich Stabilität überlegen, und ihre primäre Verwendung sollte bei Risikopatienten erwogen werden – bei Revisionseingriffen können sie als Standard gelten. Auch im eigenen Vorgehen wurden ausschließlich Plattensysteme für die Revisionsstabilisierung bei 44 Patienten eingesetzt, lokal wurden Spongiosa vom Beckenkamm und PRP miteingebracht. Die Kombination hatte sich zuvor bei der knöchernen Defektrekonstruktion im Stütz- und Bewegungssystem bewährt. Die Freisetzung und Aktivierung von Wachstumsfaktoren aus den Thrombozyten (Superfamilie der „platelet derived growth factors“ [PDGF]) beschleunigt die Organisation des „Frakturhämatoms“, die Angiogenese und darüber die Wundheilung [39–41].

Die Notwendigkeit einer stabilen Osteosynthese bei TSWI ist nicht unumstritten und wurde durch die Ergebnisse von Francel und Kouchoukos [23, 42] infrage gestellt: Bei 151 Patienten führten die Autoren nach dem Débridement eine Rekonstruktion mit unterschiedlichen Lappenplastiken durch. Ihre Erfolgsrate betrug 94 % gegenüber 65 % einer Vergleichsgruppe von 20 Patienten mit Reverdrahtung. Im Einjahres-Follow-up gaben die Patienten mit einer Lappenrekonstruktion trotz unterlassener Osteosynthese in nur 14 % der Fälle typische Beschwerden, die symptomatisch auf eine Instabilität im Sternum hinwiesen, an. Die postoperative Überprüfung der Lungenfunktion nach 3 Monaten zeigte in dieser Studie Werte wie vor der Operation. Weiterhin konnte nachgewiesen werden, dass 98 % der Patienten ihr vorheriges Hobby wieder aufnahmen; es kehrten 79 % in ihren vorherigen Beruf zurück. Nach Ansicht der Autoren besteht kein Anlass zur Befürchtung, dass ein instabiles Sternum nach erfolgreicher Lappenplastik relevante Probleme generiert.

Diese Ergebnisse wurde von der Arbeitsgruppe um Weinand et al. [11, 32], die im Rahmen der lappenplastischen Deckung bei vollständig fehlendem Sternum auf eine additive Osteosynthese verzichteten, bestätigt. Im eigenen Patientengut wurde immer eine Reosteosynthese durchgeführt, mit ausschließlicher Verwendung von Plattensystemen.

### Prävention der tiefen sternalen Wundinfektionen nach kardiochirurgischem Eingriff

Im Hinblick auf die bedrohlichen Konsequenzen einer nicht adäquat beantworteten TSWI kommt der Prävention eine besondere Bedeutung zu. Erfolgversprechende Ansätze bilden eine intensivierte Einstellung der Blutzuckerspiegels [43, 44], die präoperative Eradikation bei nasalem MRSA-Nachweis durch lokale Anwendung z. B. von Mupirocin und der Verzicht auf einen Nikotinkonsum [11].

Es stellt sich weiter die Frage, ob die operative Technik die Inzidenz der TSWI beeinflusst. Die Entnahme der A. mammaria interna reduziert die peristernale Durchblutung um 25,7 % [45]. Die beidseitige Verwendung der A. mammaria interna stellt daher einen signifikanten Risikofaktor für die Entstehung einer TSWI dar [1, 46]. In der eigenen Kohorte wurde in 9,3 % der Fälle beidseits die A. mammaria interna verwendet. Somit ist die Forderung nach einer alternativen, das Sternum nichtskeletierenden und thermonekrotisierenden Präparationstechnik grundsätzlich gerechtfertigt, kann aber durch die vorgestellten Ergebnisse nicht statistisch belegt werden.

Vor dem Hintergrund der verbesserten Gewebsdurchblutung durch die NPWT ist ihr prophylaktischer Einsatz in Betracht zu ziehen, wie es in den Konsensus-Empfehlungen von Dohmen et al. 2014 zumindest bei allen Risikopatienten empfohlen wird [47].

Zur Verringerung resp. Stillung einer diffusen Blutung aus den spongiösen Osteotomieflächen des Sternums wird in der Herzchirurgie noch immer regelmäßig Knochenwachs eingesetzt [48]. Letzteres wird auch langfristig nicht resorbiert und stellt eine zusätzliche Beeinträchtigung der Knochenheilung dar. Es stehen inzwischen Hämostatika für die topische Anwendung am Knochen zur Verfügung, die sich in der Traumatologie und Orthopädie hinreichend bewährt haben und mit dem physiologischen Heilprozesse nicht nachhaltig negativ interferieren [49].

Kieser et al. implementierten in ihrer Studie mehrere Präventivmaßnahmen bei Risikopatienten und konnten einen signifikanten Rückgang der TSWI-Rate beobachten [50]. Serraino et al. [51] gelang 2013 durch die lokale Anwendung von PRP eine signifikante Senkung der TSWI-Rate im Vergleich zu fehlender PRP-Anwendung. Schließlich ist die Verwendung eines speziellen Sternumkorsetts bei Risikopatienten, mit der die Raten der Sternumdehiszenzen und TSWI signifikant gesenkt werden konnten, sinnvoll [52].

## Fazit für die Praxis

- Die tiefe, sternale Wundinfektionen (TSWI) ist eine seltene, dabei ernste Komplikation nach kardiochirurgischen Eingriffen mit medianer Sternotomie.
- Werden präoperativ prädisponierende Risikofaktoren identifiziert, besteht nachweislich ein Präventionspotenzial in der operativen Technik und dem perioperativen Management.
- Kommt es zu einer sternalen Wundheilungsstörung oder Instabilität, ist ein fehlender, mikrobiologischer Keimnachweis kein sicheres Negativkriterium für das Vorliegen einer bakteriellen Infektion.
- Negative pressure wound therapy (NPWT), resistogrammgerechte Antibiotikabehandlung, spezielle Sternumplattensysteme und defekt-/situationsadaptierte etablierte Lappenplastiken in einem befundadaptierten Algorithmus gehören zum Armamentarium im interdisziplinären Vorgehen.
- Durch differenzierte Lappenplastiken kann selbst der Totalverlust des Sternums erfolgreich durch eine stabile Narbenbildung kompensiert werden.
- Essenziell für den Erfolg ist ein interdisziplinär gelebtes, strukturiertes und situationsadaptiertes Behandlungsregime.
